# Mismatch Negativity: Translating the Potential

**DOI:** 10.3389/fpsyt.2013.00171

**Published:** 2013-12-18

**Authors:** Juanita Todd, Lauren Harms, Ulrich Schall, Patricia T. Michie

**Affiliations:** ^1^School of Psychology, University of Newcastle, Callaghan, NSW, Australia; ^2^Priority Research Centre for Brain and Mental Health, University of Newcastle, Callaghan, NSW, Australia; ^3^Schizophrenia Research Institute, Darlinghurst, NSW, Australia; ^4^Hunter Medical Research Institute, Newcastle, NSW, Australia; ^5^School of Medicine and Public Health, University of Newcastle, Callaghan, NSW, Australia

**Keywords:** mismatch negativity, auditory event-related potential, schizophrenia, NMDA, synaptic plasticity, NMDAR

## Abstract

The mismatch negativity (MMN) component of the auditory event-related potential has become a valuable tool in cognitive neuroscience. Its reduced size in persons with schizophrenia is of unknown origin but theories proposed include links to problems in experience-dependent plasticity reliant on *N*-methyl-d-aspartate glutamate receptors. In this review we address the utility of this tool in revealing the nature and time course of problems in perceptual inference in this illness together with its potential for use in translational research testing animal models of schizophrenia-related phenotypes. Specifically, we review the reasons for interest in MMN in schizophrenia, issues pertaining to the measurement of MMN, its use as a vulnerability index for the development of schizophrenia, the pharmacological sensitivity of MMN and the progress in developing animal models of MMN. Within this process we highlight the challenges posed by knowledge gaps pertaining to the tool and the pharmacology of the underlying system.

With more than 150 papers discussing or reporting smaller mismatch negativity (MMN) amplitude in schizophrenia, changes in this component of the auditory event-related potential (ERP) are now recognized as one of the most replicable electrophysiological abnormalities in this group. This review begins with an explanation of why this finding holds so much potential as a tool in the study of biological changes associated with the schizophrenia illness. However, the sections that follow expose the many challenges to the endeavor to translate this research – both in terms of understanding the meaning, relevance, and cause of smaller MMN amplitude and in terms of building animal models that can provide insight into etiology.

## The Potential – Why is There so Much Interest in MMN in Schizophrenia?

Auditory MMN is evident in scalp-recorded evoked potentials when an unexpected event or sound transition occurs in a regular repeating pattern (1). MMN is not a response to novelty *per se* but rather to how unlikely a particular sound transition is given a preceding sequence (2). It is therefore a very context-dependent evoked potential that only occurs when a pre-existing *prediction-model* exists specifying the most likely sound transitions in the present environment.

The nature of sound sequences used to elicit and study MMN range from very simple to highly complex. The vast majority of studies in schizophrenia employ the former in which a regular repeating identical sound occurs with high probability (the *standard*) and a physically deviant sound (the *deviant*) interrupts these repetitious trains on rare occasions (estimates suggest max *p* ≤ 0.30) (3). Sequences of this kind, known as oddball sequences, promote the formation of a prediction-model specifying that acoustic input is best explained by standard-to-standard transitions and the rare occurrence of a standard-to-deviant transition is generally used to index MMN (although note that deviant-to-standard transitions also elicit a smaller MMN-like response). Regularities are implicitly learned as MMN does not require attention to sounds: sound sequences are usually presented via headphones to participants who are asked to ignore sounds and direct attention to an alternate task (3).

The classical derivation of MMN involves a deviant-minus-standard difference waveform with MMN quantified as the most negative peak evident between ~100 and 250 ms following the point of deviance (4). The quintessential finding in studies of this kind is that the averaged response to standard stimuli is similar in schizophrenia and matched control groups but the response to the deviant is significantly smaller resulting in smaller MMN in the difference waveform (5).

The major cortical sources contributing to scalp-recorded MMN are located bilaterally in primary and secondary auditory cortices with the precise locations dependent upon the sound characteristics (6). Intra-cortical recordings in primates suggest that the additional negativity in scalp-recorded response to deviations has its origin in lamina II/III of the auditory cortex (7). The neural mechanisms contributing to the difference in response to predicted versus deviating sound are the topic of considerable debate. There is general acceptance that processes such as neuronal adaptation make an important contribution; that is, the regular stimulation of the same afferent pathways will result in adaptation which reduces the response to sounds matching these properties, while in contrast deviating tones that are physically different will stimulate new afferent pathways (8–10). However, both sophisticated sequence designs and computational modeling support the existence of additional processes subserving prediction. Although some of these will be reviewed in more detail below (see The Measurement of MMN – Is there an Optimal Paradigm with Which to Study MMN in Schizophrenia?), extensive discussions can be found elsewhere (2, 3, 11–13). In summary, there is accumulating evidence that the altered responsiveness to sounds (reduced to expected and sensitized to deviant) cannot be explained by neuronal adaptation alone. Computational models suggest that predictions are expressed in the input from higher to lower levels within a hierarchical network (11, 12, 14). Changed response to sound therefore becomes a function of both neuronal adaptation and top-down inputs to auditory cortex (e.g., from secondary to primary cortex and from prefrontal to secondary cortex) that modify responsiveness to sound reflecting the predicted continuation of a learned pattern.

A network-level appreciation of MMN generation is valuable because it emphasizes that understanding the smaller MMN amplitude in schizophrenia could require consideration of both cause and consequence, acknowledging the functional role of the signal. According to free-energy conceptualizations of brain function, prediction-modeling (the use of regularity to extrapolate patterns) provides a regulatory service allowing the brain to conserve energy (15). Reduced response to the predicted causes of sensory stimulation, such as diminished response to standard input, reserves resources for events that contradict expectations and that may signal important changes in the environment prompting new learning. MMN signals a *prediction-error* indicating that the model presently influencing cortical response has failed to account for the current sensory event. The error communicates a need to adjust the current model to facilitate more accurate predictions, and simultaneously alerts the system to the possible importance of the eliciting event (15, 16). The error is communicated upward (via feed-forward connections) within a hierarchical network which enables future predictions to be weighted by error frequency (17). When a model is highly reinforced (low error frequency) predictions specified in top-down connections are weighted strongly and errors elicit large MMN. Conversely, when error frequency is higher the weighting on top-down predictions is reduced and MMN elicited by errors is smaller in amplitude. This concept has elsewhere been described as *predictive confidence* in a model which is of course inversely related to the probability of deviations (2). MMN amplitude therefore reflects quantification of confidence that the eliciting event violates a contextual regularity. Large MMN can trigger an orienting response with consequences for performance of concurrent tasks (18–23). These observations demonstrate the way in which a deviation from predictions can draw upon resources engaged in other activities.

In schizophrenia, MMN as a process appears to be largely intact in that it obeys similar principles to those evident in healthy comparison groups – MMN is larger for deviations that are rarer and/or more physically different from the current prediction-model (24). However, MMN amplitude reaches asymptote at a lower amplitude resulting in the most pronounced group differences generally being observed where MMN is very large in controls (25–27). Understanding the functional relevance of MMN amplitude is pivotal to understanding the potential impact of smaller MMN in schizophrenia. It has been proposed that MMN amplitude in part communicates the change in cortical responsiveness required to accommodate the new event into prediction-models (16). To the extent that this is true, the lower plateau in MMN amplitude in schizophrenia means that the adjustment to predictions prompted by larger or rare deviations may be equivalent to that for smaller or more frequent deviations. So if the size of the error-signal itself influences the adjustment in predictions, then equivalence of error signals across a range of deviant probabilities or physical differences (25–27) would perpetuate insensitivity to these different contexts.

This circular challenge becomes particularly relevant when considering how smaller MMN is related to biological changes associated with the illness, such as gray matter changes and involvement of the *N*-methyl-d-aspartate (NMDA) glutamate receptor (NMDAGluR) system. A key biological change observed in schizophrenia is loss of cortical gray matter volume (28–35). Within the auditory system this volume loss is due to a combination of reduced pyramidal somal cell size, reduced dendritic spine density, and (a correlated) reduction in axon terminal density in lamina III (36–39). Molecular-level studies in schizophrenia therefore place a core pathological process in the very same cortical layer implicated in the generation of MMN. The projected functional consequence of reduced volume in this cell layer is a net “diminished excitatory synaptic connectivity” consistent with an impact on the spread of activation [(36), p. 384]. The generation of MMN includes spreading activation across lamina III within A1 (Heschyl’s gyrus or primary auditory cortex) but also importantly from A1 to STG [superior temporal gyrus or auditory association cortex (11, 17)]. So in terms of MMN generation, this pathology in auditory cortical regions is expected to lead to smaller MMN generated in response to error and/or a lower limit to MMN size [consistent with experimental observations reviewed (36, 38)]. This predicted consequence is also consistent with observations that in schizophrenia (but not in matched controls) limited MMN size correlates with reduced gray matter volume in auditory regions (35, 40) and in the only published longitudinal study, progressive loss of gray matter in auditory cortices correlated with progressive reduction in MMN (40).

The etiology of this auditory cortical pathology in schizophrenia is unclear but two hypotheses put forward include a developmental origin and/or failure in sustained support of structural integrity (36). The first supposes that there is an over-elimination of excitatory synapses in lamina III during development. Since this process is very protracted [continuing into the third decade in auditory cortices (41)], it maps well onto data showing progressive reductions in STG gray matter volume in schizophrenia over this period (42). However, experience-dependent plasticity modifies dendritic spine structure throughout life (through long term potentiation and depression) and this activity has a stabilizing effect on these structural elements. The same pathology could therefore also arise (uniquely or in conjunction with developmental over-pruning) through failure in the factors that should support structural integrity.

The second and not unrelated biological factor with relevance for smaller MMN in schizophrenia is the impairment in NMDAGluR system function proposed in glutamatergic models of schizophrenia which have become increasingly accepted as etiopathological models of schizophrenia. These models arose from observations that the drug phencyclidine (PCP) has (i) psychotomimetic effects and (ii) non-competitively blocks NMDAGluRs [for detailed reviews see Ref. (43–45)]. NMDAGluRs have a key role to play in experience-dependent synaptic plasticity, and in particular long term potentiation and depression. Importantly (as reviewed later in Section “How do Pharmacological Manipulations Alter the MMN Process?”), MMN is reduced in healthy individuals administered an NMDAGluR antagonist, such as ketamine. So the question that emerges is this: is smaller MMN in schizophrenia a consequence of gray matter reduction and/or NMDAGluR hypofunction in auditory cortex, or could the process of prediction-modeling in audition provide clues as to why structural integrity in this region is inadequately supported? In summary, the reasons to suppose that MMN can be used as tool to provide insights into schizophrenia-related brain changes are many and derive from both empirical and theoretical origins. In the following sections we review MMN research from several perspectives to identify some important challenges to realizing its potential in the study of biological processes linked to schizophrenia.

## The Measurement of MMN – Is There an Optimal Paradigm with Which to Study MMN in Schizophrenia?

Smaller MMN in schizophrenia has been observed over a number of different experimental paradigms so the underlying reasons for MMN-reduction are likely to reflect the common demands inherent in processing the various sound sequence types. Different paradigms each have advantages and disadvantages (reviewed below) and it is the authors’ opinion that there is no ideal paradigm. In fact, to nominate an ideal could be detrimental to the field – the advantage in doing so (increased comparability between studies) is outweighed by the disadvantage which is neglect of the opportunities to use this tool to address specific questions about the integrity of the underlying system.

### Considerations in measuring a difference waveform

There are at least three contributing factors to MMN obtained in the classical deviant-minus-standard waveform: (i) differences in physical attributes of the deviant and standard sounds (this is particularly likely when the deviant contains physical features that vary in acoustic energy such duration or intensity), (ii) differences in refractoriness (or more properly called adaptation) of the neural generators of responses to the more frequently presented standard versus rare deviant stimulus and (iii) a genuine deviance-detection process or the *true MMN* resulting from the deviant violating predictions derived from contextual regularities. This means that a group difference in classically derived MMN could arise from any one or a combination of these factors. A number of experimental control protocols have been developed to refine a measure of *true MMN* – the primary motivation for these studies being to determine whether a true MMN exists or whether what is measured as MMN could be accounted for entirely by adaptation effects on other components of the ERP. In a series of papers, Schröger and his colleagues devised a random control stimulus sequence, the *many-standards* sequence. For example in the case of a frequency deviant, a many-standards control would involve random presentations of tones of different frequencies (including tones with the frequencies of the deviant and standard tone) each having the same probability as the deviant in the oddball sequence [Frequency/pitch: (46–48); Duration: (49); Location: (50)]. A comparison of responses to the deviant from an oddball sequence and to the same sound in a many-standards sequence controls for physical differences and adaptation contributions to the classically derived MMN. In this way the resultant difference waveform can be attributed to a novel event violating a *stored neural representation* of regularity in recent stimulation. This is because the random presentation order of the many-standards sequence *prevents the development of a representation of regularity in recent stimulation* but preserves the rate of occurrence and therefore some level of equivalence in terms of adaptation effects.

To our knowledge there are no studies that have implemented conservative control procedures to extract true MMN in schizophrenia. As noted above, the vast majority of the published literature in schizophrenia has employed MMN derived using the classical oddball derivation. The common finding of similar responses to standards in schizophrenia and matched control groups is reassuring in that it suggests that adaptation effects on the standard response at least are not detectably impaired in patients and implies that the major group differences are in how the brain responds to deviations. However whether there are group differences in the extent of cross adaptation to other frequencies (or durations/intensities, etc., in the case of simple deviance paradigms) is unknown and this could impact on the response to a physically different deviant tone. It is also reassuring to note that in healthy individuals, even when stringent controls such as the many-standards control are implemented, the classical derivation gives a reasonable approximation of the true MMN (49). But once again – whether this is equally true in patients is unknown. Below we review some of the literature employing non-classical MMN derivations indicating that there may indeed be some group differences in response to repetitive sound as well as the response to deviations.

### Examples of paradigms used in schizophrenia

A few patient studies have employed methods that can differentiate components of the MMN process and these have yielded mixed findings. Some have controlled for the physical differences between standard and deviants sounds, either by presenting two oddball sequences with the roles of deviant and standard sounds reversed [a flip-flop design: (51, 52)] or by presenting a sequence consisting of repeated presentations of deviant sounds only (53), referred to as a deviant-as-standard sequence. In both instances, it is possible to derive difference waveforms from comparing responses to the *same sound* as a repetitive standard and a deviant event. While these studies report no group difference in response to the repetitive oddball standard presentations, Todd et al. (54) recently found evidence of clear group differences in the morphology of ERPs to the repetitious presentation of deviants and these differences had a significant impact on computation of the MMN. The group difference appeared in a negative component of the ERP to repetitive sounds occurring ~200–300 ms post-stimulus. While its origin and functional role are unknown, it suggests that such differences in cortical response to a repetitive sound more generally warrant further investigation. However, neither the flip-flop control nor the deviant-as-standard sequence removes the contribution of adaptation effects to the difference waveform. Nor does it ensure that the computed MMN represents true deviance detection of a violation of contextual regularity. That is, these procedures do not allow the extraction of the *true MMN*.

The roving paradigm is another method that can partially address group differences in various contributions to MMN. In a typical roving paradigm a string of sounds of the same pitch are eventually interrupted by a sound with a higher or lower pitch which then continues to repeat. Predictive processing is highly dynamic so within two to three repetitions, the new sound becomes a standard and deviations from its properties will now elicit MMN (55–57). In the roving paradigm, it is possible to study the way the response to a new standard changes after incremental repetitions (e.g., 6 vs. 12 vs. 24, etc.). An increasing positivity as a function of repetition length, termed “repetition positivity,” is apparent in the ERP to standards approximately 50–200 ms post-stimulus. The increase in MMN amplitude with the length of repetition is referred to as a memory trace effect and is a function of both this apparent increased positivity (which is in fact a decrease in negativity) to the standard and increased negativity to a subsequent deviant [although see Ref. (58)]. In schizophrenia, the increment in both components is smaller than that in controls (59), but more notably in this study, the positivity in response to standard repetitions failed to increment at all. So in this paradigm smaller MMN in schizophrenia appears to indicate less change in responsiveness to sound generally.

In a recent magnetoencephalographic (MEG) study in schizophrenia (60), the MMN_m_ data in a similar roving paradigm was explored using a technique called dynamic causal modeling (DCM). DCM differs from conventional source modeling and brain connectivity methods by utilizing a biologically informed causal model placing constraints on model inversion such that the parameters of reconstruction describe specific processes like change in synaptic coupling strength between source locations and postsynaptic gain. Rather than estimating dipole activity at a particular point in time; it models dipole activity over a period of time to identify parameters that change (11). When applied to the roving paradigm, DCM has provided evidence supporting the conceptualization of MMN as an active contextual perceptual inference process. The “best fit” to experimental data is achieved by a model that incorporates both local intrinsic adaptation effects as well as plastic changes in extrinsic inputs to auditory cortex (i.e., from STG to A1 and also from areas of the prefrontal cortex to STG). When applied to schizophrenia data, DCM has provided evidence for problems in two of three components: the largest effect size for group differences was for reduced change in intrinsic connections within primary auditory cortex A1. Such changes are considered evidence of impaired feature specific adaptation. There was also a reversed polarity in changes to connectivity between prefrontal and auditory areas which was interpreted as a failure in the normal influence of these top-down inputs in modifying auditory cortical response. The authors also comment on reduced modulation of the forward connection from the A1 to the STG (but this was not significant according to Table 2, p. 26). Although this is the first study of its kind in schizophrenia, the results conform to the view that impaired signaling of error (smaller MMN) could be indicative of impairment in encoding the contextual memory against which deviance is registered (potentially including impaired adaptation), but also consequently impaired ongoing modification of cortical responsiveness by feedback projections. It is therefore possible that the roving standard paradigm is more sensitive to any problems in forming a contextual memory based on tone repetitions. However, when interpreting results it is important to consider the assumptions of the roving paradigm carefully before drawing this conclusion. The MMN elicited to a change in frequency in a roving paradigm signals that the current prediction-model failed to account for the present stimulus properties and the model may require updating. With repetition of the new frequency, an updated prediction-model is built. The degree to which the model requires updating will be a function of the difference between the representation of the new and former standard frequencies. Given that the changes in frequency can be quite subtle and that frequency discrimination is impaired in schizophrenia (53, 61, 62), it is possible that less evidence of updating after a new standard (repetition positivity) could in part reflect less distinct representations of the new and former frequency. So the roving paradigm places considerable weight on stimulus-specific adaptation to a new frequency, which therefore may augment the importance of intrinsic adaptation in A1. Whether DCM of a classic oddball paradigm would replicate major group differences in intrinsic A1 connections remains to be determined.

At present the literature certainly suggests that altered (smaller) response to sequence deviations is a major contributor to smaller MMN in schizophrenia. It is clear that the reduction in classical MMN is not only robust across many different cohorts, laboratories, ethnic groups, etc. (5), but also exhibits substantial stability over time (63). However there is reason to suppose that there may also be differences in responding to repetitive sound more generally that could be contributing to problems in prediction-modeling and/or MMN computation. Novel paradigms and novel data processing approaches are likely to provide valuable data with which to address these contributions. Despite these differences it should be noted that many elements of the predictive process underlying MMN remain intact in schizophrenia. In addition to those covered above we recently demonstrated that persons with schizophrenia, despite producing smaller MMN amplitude to deviants, are equally able to *reduce* MMN size to a deviant if the occurrence of that deviant could be inferred from the identity of the prior tone. The equivalent use of this predictive information in both schizophrenia and control group reinforces the position that abnormalities in the MMN process in schizophrenia primarily involve limited gain in the differential response to a very rare versus common event. So while the choice of paradigm used to study MMN in schizophrenia will depend not only on the questions driving your research but also the time you have available, the one principal recommendation that we do put forward is to ensure that you adopt a very rare deviant event (<15%) to maximize your power to expose group differences.

## Does MMN-Reduction Indicate Vulnerability to Schizophrenia?

In this section we review studies addressing whether small MMN may be a vulnerability index for schizophrenia. It should be noted that research into schizophrenia has always been limited by its diagnostic heterogeneity and phenomenological overlap with related developmental, affective, or personality disorders which all can share some of the clinical features of schizophrenia, such as psychosis, cognitive impairment, or therapeutic response to certain classes of pharmacological agents (64). Not surprisingly, attempts to find a diagnostic marker for schizophrenia – that not only represents an *endophenotype* of the disorder but can also serve as a tool helping to unveil its pathology – continues to be limited by the lack of a pathogenomic definition of the condition. Diagnostic limitations notwithstanding, a number of groups have indeed investigated MMN’s status as an endophenotype for schizophrenia as defined by current diagnostics tools and this research is pertinent to evaluating the translational potential of MMN in schizophrenia. The definition upon which the evaluation is based is that proposed by Gottesman and Gould (65) where an endophenotype is defined as an intermediate phenotype along the pathway between genotype and the observable established aspects of the illness. Specific criteria to be met include: (i) it is associated with illness in the population; (ii) it is heritable; (iii) it is primarily state-independent (manifests in an individual whether or not illness is active); (iv) within families, small MMN and the illness co-segregate; and (v) smaller MMN is evident in non-affected family members at a higher rate than in the general population (65).

### Criterion (i) and (ii)

Mismatch negativity amplitude reduction in schizophrenia is a very robust finding with an effect size of 0.99 observed by Umbricht and Krljes (5) in their meta-analysis of 32 studies published prior to 2004. Reduced MMN is less prevalent in related conditions, such as bipolar affective disorder independent of the presence of psychotic symptoms (52, 66–68) and in major depression (66) but see (69–71). That is, there is considerable evidence that criterion (i) is met in terms of its association with the illness, although its specificity to schizophrenia is less clear than initially thought. MMN appears to be heritable based on twin studies with heritability estimates ranging from 0.48 to 0.68 when using a duration increment deviant (67, 72) but not for a frequency deviant (73). However, even duration MMN shows only weak phenotypic association (0.39) with schizophrenia (67). Nonetheless, there is preliminary evidence supporting criterion (ii) for endophenotypic status of MMN.

### Criterion (iii)

Across the large number of studies on MMN in schizophrenia there are no consistent relationships between MMN size and the severity of symptoms of psychosis. Although impaired prediction-error signaling is implicated in the genesis of delusions (74) and although there is preliminary but consistent evidence from one group of a relationship with auditory hallucinations (75–77), the literature fails to demonstrate consistent relationships across studies. The meta-analysis by Umbricht and Krljes (5) emphasized that the majority of studies did not find correlations (either with positive or negative symptoms) and observed no change in MMN when symptoms improved. Further (63), found duration MMN in very large sample of patients (*N* = 163) exhibited substantial stability across a 1 year retest interval, and to be independent of fluctuations in clinical symptoms, positive or negative. So at face-value, these results from cross-sectional designs seem to support state-independence of presence of smaller MMN [criterion (iii)]. However, the evidence about state-independence of degree to which MMN is reduced in patients in an acute phase vs. a post-acute phase is mixed (78, 79) although differences due to medication changes cannot be eliminated (see later Section “How do Pharmacological Manipulations Alter the MMN Process?” for further discussion of state vs. trait effects on MMN as a vulnerability marker).

The failure to demonstrate state-dependence is paralleled by more consistent relationships between MMN amplitude and relatively stable features of the illness such as level of functioning (80) and cognitive impairments (81) despite substantial changes in negative and positive symptom severity. Various measures of current functioning have been shown to be associated with MMN amplitude in patients: global assessment of function [GAF: (82–84)], social and occupational functioning assessment scale [SOFAS; (35) but not in first episode patients (85)], the independent living scales [ILS: (86)], and work functioning and independent living ratings from the role functioning scale (87). It has been suggested that the relationship between reduced MMN and impaired functioning might be mediated by anatomical changes such as gray matter loss in relevant brain regions (35). Impaired cognition (whether independent of gray matter declines or consequential) could also mediate the relationship between MMN and functional status since there is a wealth of evidence now that the strongest predictor of functional outcomes in patients is cognition [also often called neurocognition: (88)]. However, the number of published MMN studies in schizophrenia that examine not only cognition in the same sample but correlations between MMN and cognitive performance as well, is quite limited. This is despite reasonable expectations that the reliance of MMN generation on the the NMDAGluR system (see How do Pharmacological Manipulations Alter the MMN Process? and Translation to Animal Models below for further discussion) should lead to relationships with those aspects of cognition that are also reliant on the unique characteristics of the NMDAGluR, such as context-dependent effects, integration of information over time and new learning (44). However, there are some data that suggest such relationships do exist at least in patients (but not necessarily in healthy controls).

NMDAGluRs have a number of unique features. Firstly, activation of NMDAGluRs currents is conditional in that channels only gate following presynaptic release of glutamate and concurrent postsynaptic membrane depolarization which relieves Mg^2+^ blockade. This conditional characteristic of the NMDAGluR is likely to be particularly important where responses are determined by context, as is the case for MMN, but also in situations where flexibility of response is required dependent on context. One specific example of contextual processing is the AX version of the continuous performance (AX-CPT) task where a response to the letter X on screen should only executed when the X is preceded by the letter A. It is well established that patients are impaired on the AX-CPT task, producing often fewer correct responses to AX sequences (impaired priming of response by a target-consistent cue) and higher rates of false alarms to BX sequences [impaired inhibition of a response prompted by a target-inconsistent cue (89)]. However, despite reports of concurrent smaller MMN and AX-CPT impairments, there appear to be no reports of a correlation between the two (90) and one explicit report of no association between the two (54).

Secondly, although NMDGluRs exhibit complex kinetics with evidence of multiple gating modes characterized by different mean open times (91–93), it is generally accepted that they mediate long duration excitatory postsynaptic currents in the brain and participate in synaptic integration and certain forms of synaptic plasticity (92). Prefrontal cortex NMDAGluRs in particular have slower kinetics than sensory regions (94) and therefore are potentially involved in maintaining activity in prefrontal neurons (95), for example during the delay periods of working memory tasks. There are reports of co-occurrence of working memory deficits and reduced MMN in patients with schizophrenia (63, 90) but to our knowledge, only one report of a correlation between working memory (measured using the digit sequencing task from the Japanese version of the brief assessment of cognition) and (duration) MMN in patients (96). Both classic oddball MMN amplitude (97) and longer term effects on growth in MMN amplitude (98) have been shown to correlate with digit span (the ability to maintain and or manipulate acoustic presentation of digits) in a healthy control group consistent with the requirement to store auditory information over time for both indices.

Thirdly, NMDAGluR activation leads to a cascade of events that initiate long term potentiation and depression, the primary processes responsible for new memory formation and learning in hippocampus and cortex. Retention or storage of information is less reliant on NMDAGluR. One of the most robust cognitive deficits exhibited by patients are deficits in memory (verbal declarative memory in particular) with the majority of evidence suggesting that the largest deficit, as measured by effect size, occurs for encoding or new learning of material in comparison to retention [although there is still a small deficit in retention even when initial learning differences are taken into account (99)]. Four studies report relationships between MMN and memory performance but in patients only. Baldeweg et al. (59) using a roving oddball paradigm found that the MMN trace effect (the increase in MMN that occurs with increasing repetition of prior standards) correlated with performance on an everyday memory test (Rivermead behavioral memory test). Kawakubo et al. (100) in a study that reports data on patients only found that MMN elicited by a phoneme duration deviant (but not a tone duration deviant) was correlated with immediate free recall (initial learning or encoding measure) from a list learning task, the Rey auditor verbal learning task (RAVLT). In contrast, Kaur et al. (101) found that tone duration MMN correlated with verbal memory assessed using the RAVLT in first episode psychosis patients with a schizophrenia spectrum diagnosis of either schizophrenia, schizoaffective, and schizophreniform illness. No correlations were reported for controls. Kiang et al. (84) in a standard oddball paradigm found duration MMN was correlated in patients only with short-delay as well as long delay free recall but not (significantly) with immediate free recall on another list learning task, the California verbal learning task, but neither of the free recall measures was adjusted for initial or prior learning differences. So while there is evidence of MMN correlations with memory, these correlations may not be restricted to initial learning or encoding phase.

In addition, there is evidence of relationships between MMN amplitude and other cognitive domains that are commonly shown to be impaired in patients but are less clearly dependent on NMDAGluR properties, such as executive functions [anti-saccade performance: (102); proverb interpretation: (84); perseverative errors on the Wisconsin Card Sorting Task: (103); mental control subtest of WMS-III: (85)].

In summary – MMN does not appear to be consistently related to the severity of either positive or negative symptoms experienced by the patient at the time of recording (either examined across patients or assessed within patients at different time points), consistent with (symptom) state-independence of MMN. However, MMN amplitude does appear to vary across individuals as a function of more stable features of their illness. The relationship of MMN to functional measures is relatively robust and there is growing evidence of correlations not only between MMN amplitude and those aspects of cognition that are likely to be reliant on unique aspects of NMDAGluR but other domains of cognition that are reliably impaired in patients such as executive functions. However, these are issues that deserve more attention and in particular, more systematic investigations are required before any strong assertions can be made about contributions from a common mechanism of NMDAGluR-dysfunction to MMN-reduction and cognitive deficits in schizophrenia.

### Criterion (iv) and (v)

Mismatch negativity has also been investigated as a potential predictor of developing psychosis or schizophrenia in populations considered at-risk mental state (ARMS). For instance, the comprehensive assessment of at-risk mental state (CAARMS) criteria (104) defines ARMS as (1) a significant drop of global functioning over a period of 12 months and having a close biological relative with a psychotic disorder and/or (2) experiencing attenuated or very brief episodes of psychotic symptoms in combination with functional decline. MMN has been investigated in both groups; however, to date there appear to have been no MMN longitudinal studies of transition to psychosis specifically within a clinically unaffected genetic group.

Whether MMN is reduced in unaffected first-degree biological relatives of patients with schizophrenia is controversial. The initial studies by Jessen et al. (105)^1^ and Michie et al. (106) reported reduced oddball frequency and duration MMN respectively in first-degree relatives when compared to healthy controls, but these results with one exception were not replicated in later publications, neither for duration deviants – (72, 107–109) and (102), [based on a larger sample from the same source as (106)], nor frequency deviants – (73, 108, 109). The one exception is (110) which interestingly used an identical MMN duration deviant paradigm as (106). While there are design and other methodological differences between those studies (three in total) that show significant MMN-reductions in first-degree relatives and those that do not (six in total), the bulk of the evidence suggests that at-risk but clinically unaffected family members do not exhibit reduced MMN (minimal support for criterion (iv) and (v) of endophenotype criteria).

The evidence that MMN is reduced in the ARMS group, is more consistent, although data on whether reduced MMN predicts transition to psychosis is still preliminary. It is important to note that while the term *prodromal* is sometimes used to describe those clinically defined at-risk groups (111), strictly speaking whether they are prodromal or not at the time of assessment can only be determined subsequently by whether they develop a schizophrenia spectrum disorder within the follow-up period (usually 12–24 months). All of the MMN investigations in ARMS have used duration deviants, either a duration increment (112–117), or a duration decrement (111, 118) or both (112) but some also report data on frequency deviants in the same sample (111, 116–118) or a double deviant [deviant on both frequency and duration (117)]. Of these eight papers, five found that duration increment MMN was significantly reduced in the ARMS group (112–114, 116, 117) whereas duration decrement MMN was either not significant (111, 118) or showed a smaller effect size (112). The findings for frequency MMN are mixed: (117) found that frequency MMN was reduced (as was the double deviant) but (116) did not. To date therefore, the evidence seems to suggest that deviants that differ from standards by being longer in duration are more sensitive to the at-risk mental state that other deviant types. It seems unlikely that these findings are due to the effects of medication since in each sample the numbers of ARMS individuals who were medicated with anti-psychotics at the time of testing was small as were the dosages.

Most investigations of clinical high risk groups also report transition data and examine whether MMN predicts those who will subsequently develop a schizophrenia disorder – although in two cases, the number of transitions was too small for statistical analysis (112, 113). Bodatsch et al. (118) were the first to report transition data. Interestingly they found that duration (decrement) MMN predicted those who converted to schizophrenia within a 24 month period of the assessment date whereas frequency MMN did not. Higuchi et al. (115) and Shaikh et al. (114) observed similar results for duration (increment) MMN. Neither included a frequency MMN deviant. However (117), found that the best predictor of later transition to schizophrenia was MMN to their double deviant. Neither duration alone nor frequency alone was significant. Perhaps importantly, the double deviant elicited the largest MMN (larger than that to frequency or duration alone) which perhaps reflects the importance of challenging the upper limits on MMN size in at-risk groups as well as patients with an established illness. In summary, evidence for smaller MMN within families in general is not strong but the evidence for smaller MMN in clinical high risk groups is quite compelling. Therefore endophenotype criterion (iv) and (v) are only partially met at best. It remains to be seen whether the reduction in the at-risk groupings is really about risk status *per se* or a reflection of schizophrenia-related pathology that has begun to impact on brain function.

## How Do Pharmacological Manipulations Alter the MMN Process?

Pharmacology as a field of research offers a unique avenue to study MMN both in terms of how different chemicals can perturb the perceptual inference process and how they may relate to schizophrenia pathology. When considering the pharmacological sensitivity of MMN, it is clear that a change in MMN could reflect an effect on any one of a number of constituent processes described in Section “The Measurement of MMN – Is there an Optimal Paradigm with Which to Study MMN in Schizophrenia?” Surprisingly few studies provide details on how a substance affected response to standard repetitious sounds with the majority reporting on the difference waveforms only (16/27 studies, see Tables 1–3). For the purpose of this review we have restricted Tables to acute drug effects on healthy adult populations. One of the key foci in pharmacological research on MMN is how it is affected by alterations to NMDAGluR activity (Table 1).

**Table 1:** ***N*-methyl-d-aspartate receptor studies**.

Reference	Study design	Main findings	Comments
Oranje et al. (119)	Double-blind, placebo-controlled randomized ketamine challenge (0.3 mg/kg) in 18 healthy male volunteers	Processing negativity (PN) and P3 amplitude reduced and N1 amplitude increased with ketamine; no effects observed on MMN (frequency deviants)	Ketamine did not affect error rate and reaction time in selective attention task; dose lower than in studies showing an effect on MMN; study reported ERPs in response to standard stimuli
Umbricht et al. (120)	Single-blind placebo-controlled ketamine challenge (0.9 mg/kg/h) in 20 healthy volunteers whilst performing a continuous performance task	N1 peak amplitude increase with ketamine; MMN (i.e., frequency and duration deviants) amplitude reduction with ketamine; MMN (i.e., duration deviants) peak amplitude latency increase with ketamine	MMN topography was not altered by ketamine; study did not report ERPs in response to standard stimuli
Kreitschmann-Andermahr et al. (121)	On-off ketamine (0.3 mg/kg) single-session MEG trial in 13 healthy volunteers (final sample size *N* = 10 with sufficient data quality)	Ketamine affected MMF latency and dipole moment due to effects on deviants (frequency, duration, and intensity); no effect on N1	Ketamine reduced mean global field power for MMF; study reported ERPs in response to standard stimuli
Umbricht et al. (122)	Single-blind, placebo-controlled psilocybin challenge (0.28 mg/kg) over two sessions; ERP recorded 70 min after drug administration in 18 healthy volunteers	N1 peak amplitude reduction with psilocybin; no effect on P2	Non-significant trend toward smaller MMN amplitudes for frequency deviants with psilocybin; study did not report ERPs in response to standard stimuli
Korostenskaja et al. (123)	Randomized, double-blind, placebo-controlled crossover challenge of memantine (30 mg) in 13 healthy volunteers	Trend of MMN amplitude increase in response to frequency deviants with memantine	No effect on MEG derived measures of MMN, P1 and N1; study did not report ERPs in response to standard stimuli
Heekeren et al. (124)	Randomized, double-blind, crossover ketamine of 0.007–0.2 mg/kg and dimethyltryptamine of 0.011–0.3 mg/kg challenges, with same-day (after 2 h break) single-blind low and a high-dose drug administration, respectively, in 15 healthy volunteers (9 study participants completed both drug challenges)	Reduced MMN amplitude with ketamine; no effect with dimethyltryptamine	Subjects performed a continuous performance task was performed whilst EEG was recorded; study reported ERPs in response to standard stimuli
Roser et al. (125)	Randomized, double-blind, placebo-controlled, crossover ketamine (0.5 mg/kg/h following bolus of 0.24 mg/kg) and rimonambant (20 mg) challenge in 24 healthy male volunteers	No effect of ketamine alone on MMN amplitudes (i.e., frequency and duration deviants); addition of rimonambant resulted in MMN amplitude reduction	Ketamine dose lower than in studies showing an effect on MMN; study did not report ERPs in response to standard stimuli
Schmidt et al. (126)	Double-blind, placebo-controlled ketamine challenge (0.006 mg/kg/min following bolus of 10 mg) in 19 healthy volunteers and psilocybin challenge of 0.115 mg/kg in 20 healthy volunteers	Reduced frontal MMN with ketamine with increasing number of standards (roving paradigm); no effect on MMN with psilocybin	Placebo MMN amplitudes correlated with severity of cognitive impairment induced by ketamine; study did not report ERPs in response to standard stimuli
Gunduz-Bruce et al. (127)	Double-blind, placebo-controlled ketamine challenge (a bolus of 0.23 mg/kg over 1 min followed by 0.58 mg/kg for 30 min and then 0.29 mg/kg for 40 min) with and without *N*-acetylcystein co-administration (oral doses of 2000 mg followed by 1000 mg 2 h later) in 16 healthy volunteers	MMN amplitude reduced for intensity and frequency deviants but not duration deviants; *N*-acetylcystein did not alter the impact of ketamine on MMN	MMN recorded with multi-deviant paradigm; study did not report ERPs in response to standard stimuli
Schmidt et al. (14)	Double-blind, placebo-controlled ketamine challenge (0.006 mg/kg/min following bolus of 10 mg) in 19 healthy volunteers (17 subjects entered the final dynamic causal modeling analysis)	Ketamine selectively reduced synaptic plasticity in the forward connection from the left primary auditory cortex (A1) to the left superior temporal gyrus along with MMN amplitude reduction	Ketamine effects on synaptic plasticity correlated significantly with ratings of ketamine-induced cognitive impairments; study reported ERPs in response to standard stimuli

**Figure 1:**
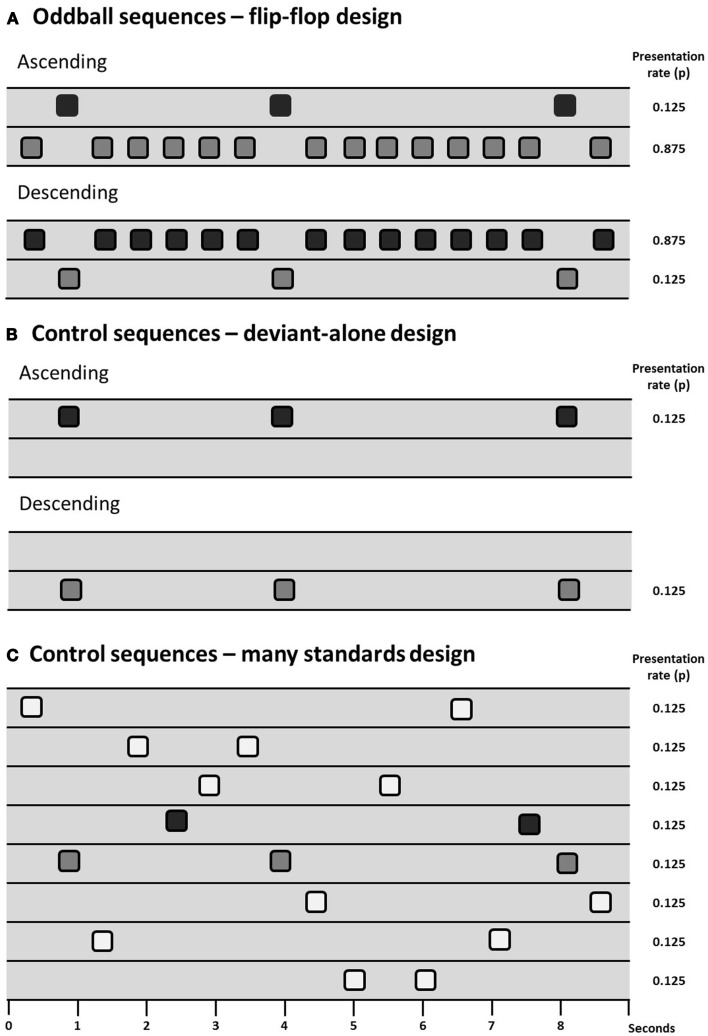
**Schematic of sequence design showing sequences with deviants presented at a probability of 0.125 (1/8), two oddball sequences are presented in a flip-flop design (A), controlling for the physical characteristics of the stimuli**. Differential adaptation can be controlled for through the use of a deviant-alone control **(B)**, which presents the deviant at the same temporal rate as it is presented in the oddball sequence, but without the intervening standards; or the many-standards control **(C)**, which presents several stimuli (including the deviant) at the same temporal rate as the oddball sequences.

Several groups have argued that impaired plasticity linked to NMDAGluRs is a core feature of the schizophrenia illness (45, 128, 129). The first study to demonstrate this link was actually in the macaque where Javitt and colleagues demonstrated a dose-dependent reduction in MMN following local infusion of the NMDAgluR antagonist, PCP (130). Using a flip-flop control design (see The Measurement of MMN – Is there an Optimal Paradigm with Which to Study MMN in Schizophrenia? above for description and Translation to Animal Models below for additional detail) the authors report no significant effect of phencyclidine on response to the repetitive sound (at least in these local field potentials, see Translation to Animal Models for further discussion) but a pronounced dose-dependent effect on the response to the deviant. The authors conclude that NMDAGluR activity is critical to forming the associative links between stimuli (i.e., accumulating information about transition statistics) that define the context against which a rare deviant sound is recognized as an aberrant event. Studies in humans are largely consistent with this initial study. Of eight published studies, seven report significant dose-dependent reduction of MMN amplitude after ketamine [although one only in combination with the CB1 inverse agonist rimonabant (125)]. The one exception (a low-dose study) used a selective attention paradigm in which participants were asked to attend and respond to stimuli in one ear while simultaneously hearing stimuli in the other ear (unattended) from which MMN was derived (119). Of the studies showing an effect of ketamine on MMN, four explicitly picture and/or discuss response to the repetitive standard tones with only one (120) reporting a slight but significant increase in the obligatory N1 component to repetitive sounds in the presence of the drug. The low-dose ketamine study by Oranje and colleagues also show enhanced N1. The most recent study reports on a roving standard paradigm demonstrating that, although MMN was reduced under ketamine overall, the effect of ketamine was more pronounced with increased repetition of the standard [i.e., when predictive confidence was highest (126)]. The paper, however, did not report on whether this pattern was due to effects on the positivity to repetitive standards or increased negativity to deviants or both. The grand-averaged (i.e., across repetitions) ERP to standards is presented in a subsequent paper reporting a DCM analysis of the same data (14). A visual comparison of Figure 1 (placebo) and Figure 2 (ketamine) from the paper suggests that the effect of ketamine on MMN was perhaps a combination of reduced positivity in the standard waveform and reduced negativity in response to the deviant. However, DCM analyses applied to the data indicated the ketamine had a selective effect on parameters representing synaptic plasticity with no effect on indices reflecting adaptation. Additionally, analysis indicated that the major effect of ketamine was to reduce the normal increase in synaptic plasticity in the forward connection between left A1 and left STG in response to the deviant tones. Therefore while ketamine administration and schizophrenia are both associated with reduced MMN, the pattern of change (at least for this roving paradigm) is quite distinct. Ketamine-induced a prominent change in feed-forward connections between A1 and STG only (14) while schizophrenia was associated principally with reduced intrinsic connections within A1 and significant alteration in prefrontal-STG connections (60).

In Schmidt et al. (14, 126), and an earlier observation by Umbricht and colleagues (131), MMN measures were associated with the intensity of psychotic-like reactions to ketamine. Schmidt et al. (126) reported that participants with the most restricted growth in MMN with increased standard repetition experienced the most pronounced disturbances noted on a “control and cognition” subscale of the altered state of consciousness questionnaire in the presence of ketamine. Similarly Umbricht and colleagues observed that the participants who produced the smallest MMNs at baseline experienced the highest symptom ratings (on a variety of measures) under ketamine. In the subsequent DCM analysis of Schmidt et al. (14), those who experienced the most pronounced “control and cognition” subscale disturbances in the presence of ketamine also showed the most pronounced decrease in plasticity in the left A1-STG connections under ketamine. Although the measures differ between studies, these observations invite the intriguing conclusion that small MMN amplitude (and/or limited growth in MMN) may indicate a limitation in synaptic plasticity that is linked to vulnerability to psychotic-like phenomena. Interestingly, this vulnerability does not appear to be generic as studies have shown no relationship between MMN amplitude and psychotic-like response to psilocybin and no significant effect of psilocybin on MMN amplitude (122, 126). Furthermore, there is evidence that this effect on NMDAGluR-mediated plasticity shows some specificity to ketamine-induced antagonism. Memantine (also an NMDAGluR antagonist) actually augmented MMN amplitude (123). The downstream effect of ketamine (and MK-801) has recently been demonstrated to be quite different to that of memantine. One interesting observation is that these compounds have opposing effects on postsynaptic density proteins – namely ketamine and MK-801 reliably increased *Homer1a* relative to *Homer1b* expression while memantine has the reverse effect. Authors suggest that the former impacts the expression of genes related to response to neuronal injury and preservation of homeostatic scaling of synaptic response. The latter, in contrast, strengthens synaptic transmission. These very different effects on plasticity may go some way to explaining the opposing effects of these NMDAGluR-antagonists on MMN and invite speculation as to whether individual differences in these same ratios may in fact confer differential susceptibility to ketamine-induced psychotic-like experiences (and disruption to the MMN process, however see also discussion of animal research using memantine in Section “Translation to Animal Models”). In summary, antagonism of NMDAGluRs in the presence of ketamine produces quite consistent reduction in MMN amplitudes and continues to hold promise in furthering our understanding of the plasticity underlying MMN as well as vulnerability to psychotic phenomena. Of course it should be remembered that acute disruption under ketamine is unlikely to mirror the full consequences of adjustment to a more chronic compromise in function (if present) in schizophrenia.

Just as there are multiple elements to the MMN process, there are multiple ways to pharmacologically influence NMDAGluR-mediated synaptic plasticity [for a relevant review see Ref. (132)]. NMDAGluR antagonism also occurs under acute exposure to alcohol and consistent with this, acute administration of ethanol has been shown to reduce MMN amplitude (133, 134). Nicotine in contrast enhances synaptic plasticity with mechanisms linked to effects on presynaptic NMDAGluRs (135). Nicotine exerts its effects on the central nervous system via acetylcholine receptors (135). Galantamine has been used to test theories about how augmentation of cholinergic neurotransmission can modulate gain in MMN (136). The results of both an empirical study and a simulation experiment indicate that enhanced cholinergic neurotransmission alters precision in prediction-modeling – i.e., changes confidence in the current inference model. More specifically, under these conditions the system places a greater emphasis on bottom-up input and “boosts” the response to deviants while also attenuating the usual reduction in confidence in a model following the occurrence of a deviance. The authors suggest that acetylcholine plays a key role in modulating gain in superficial pyramidal neurons in early sensory brain areas. Consistent with this action, five of the seven studies listed in Table 2 support nicotine enhancement of MMN. Significant enhancement of MMN has been demonstrated under both acute (137–141) and more prolonged exposure (139). Of the seven studies listed, five present or report on response to standard tones but only one indicates significant drug effects. Baldeweg et al. employed the roving standard paradigm and revealed that the increase in MMN amplitude with nicotine was due to a selective augmentation of the positivity to repeated standards with no significant effect on response to deviants.

**Table 2:** **Nicotine receptor studies**.

Reference	Study design	Main findings	Comments
Harkrider and Hedrick (137)	Single-blind placebo-controlled nicotine challenge in 10 smokers (21 mg/day) and 4 non-smokers (7 mg/day)	Larger MMN area and steeper slope in response to deviant stimuli	Small and heterogeneous sample; study reported ERPs in response to standard stimuli
Inami et al. (142)	Counterbalanced placebo versus nicotine administration (equivalent to 16.1 ± 2.7 mg/day) 10 healthy non-smokers (5 males)	Nicotine shortened MMN peak latency	Small sample size; study reported ERPs in response to standard stimuli
Baldeweg et al. (138)	Randomized placebo-controlled nicotine challenge (2 mg) in 20 healthy smokers	Nicotine increased MMN amplitude mainly by affecting response to standard stimuli (no change in response to deviant stimuli)	Nicotine enhanced repetition positivity; study reported ERPs in response to standard stimuli
Knott et al. (143)	Nicotine challenge (6 mg single dose) in 14 healthy non-smokers	No effect on MMN (frequency deviants)	Study did not report ERPs in response to standard stimuli
Dunbar et al. (139)	Randomized and placebo-controlled challenge with oral nicotine agonist AZD3480 (ascending doses from 2 to 320 mg) in 48 healthy subject and in 24 subject receiving repeatedly constant oral dose or placebo	Increased MMN amplitude with reduced latency after 10 days of consecutive agonist administration and/or single dose of 200 mg	Study did not report ERPs in response to standard stimuli
Martin et al. (141)	Single-blind, placebo-controlled nicotine challenge (4 mg) in 11 non-smokers and 9 smokers (following 2 h nicotine abstinence)	Increased MMN amplitude in response to nicotine without affecting N1 and ERPs in response to standard stimuli	Study reported ERPs in response to standard stimuli
Knott et al. (140)	Randomized double-blind, placebo-controlled crossover nicotine challenge (6 mg) in 21 non-smokers (11 males)	Drug by gender interaction of non-significant MMN amplitude increase in females and non-significant MMN amplitude decrease in males	Study reported ERPs in response to standard stimuli

Cannabis use is considered by some to be a risk factor in the development of psychosis. Furthermore, chronic cannabis use has been associated with gray matter volume changes and cognitive deficits reminiscent of those in schizophrenia (144–147). The action of endogenous cannabanoids is also linked to NMDAGluRs in protecting against excessive stimulation at glutamatergic synapses. The cannabanoids are released from postsynaptic neurons and exert their action on CB1 receptors located on presynaptic neurons which transiently decreases neurotransmitter release [(148) for review]. There are currently two published studies on the acute effects of cannabis [in fact the latter is a reanalysis of the former with genetic data included (149, 150)]. The first of these explored the effect of administering Δ9-tetrahydrocannabinol (THC) alone (the psychoactive component of cannabis) versus in combination with the other cannabinoids present in cannabis extract. The results indicated no significant impact of Δ9-THC alone, but significant augmentation of MMN at central sites in the presence of cannabis extract compared to placebo. However the study also demonstrated a significant correlation such that higher concentration of the Δ9-THC-metabolite 11-OH-THC was associated with smaller MMN amplitude (*r* = 0.62, *p* = 0.002). In their later combination with genetic data the same group revealed that susceptibility to MMN-reduction in the presence of Δ9-THC was a function of genotype for neuregulin 1 (MMN reduced in the presence of Δ9-THC for those with NRG1 rs7834206 polymorphism). Neuregulin 1 is a gene implicated in schizophrenia that influences synaptic plasticity via multiple pathways, including those involving NMDAGluRs (128). The possibility that genes confer vulnerability to Δ9-THC effects on cognitive processing also finds support in animal research where heterozygous Neuregulin 1 knockout mice have been observed to show differential sensitivity to the acute effects of Δ9-THC on behavioral phenotypes of schizophrenia (151).

Despite dopamine being a modulator of NMDAGluRs and a central focus of treatment and models of schizophrenia (37, 132), there are no studies supporting a significant effect of altered dopamine levels on MMN amplitude in healthy adults (see Table 3). Furthermore, with one recent exception (reviewed below), studies within schizophrenia do not support a significant effect of medication type or dose on MMN [see Ref. (5) for review], and no significant differences in MMN amplitude between medicated and unmedicated patients (52, 152). However, Zhou and colleagues (79) have recently reported a significant progressive increment in MMN in persons with schizophrenia treated with aripiprazole (larger at 4 and 8 weeks of treatment than at baseline). MMN was measured using a traditional oddball paradigm with two deviant types (frequency and duration) and effects are presented and reported for the difference waveforms only. Aripiprazole differs from other second generation anti-psychotics in that its action at dopamine D2 receptors shows *functional selectivity* (153–155). It has been proposed that this selectivity may be related to observations that it has differential effects on the two main dopaminergic pathways, namely a predominant effect on the mesolimbic pathway (156). Although MMN remained significantly smaller than that in matched controls, the authors argue that the effect of the drug treatment on MMN does raise questions about whether MMN amplitude is really a trait or state marker. Finally studies on benzodiazepines, often prescribed to persons with schizophrenia, have consistently failed to demonstrate any effect on MMN amplitude (see Table 3).

**Table 3:** **Monoamine receptor studies**.

Reference	Study design	Main findings	Comments
Mervaala et al. (157)	Noradrenaline challenge with alpha 2-antagonist atipamezole (0.1 mg/kg) in six healthy male volunteers	Reduced P3 amplitude without affecting MMN and Nd; improved digit span and word recognition performance	Small sample size; study did not report ERPs in response to standard stimuli
Schreiber et al. (158)	Double-blind, placebo-controlled crossover administration of ceruletide (0.5 and 2.5 mg) in 13 healthy volunteers	No effect on MMN; PN larger with ceruletide	Study did not report ERPs in response to standard stimuli
Kahkonen et al. (159)	Placebo-controlled haloperidol challenge (2 mg) in 12 healthy volunteers	MMN increased with haloperidol without affecting other ERP components	MEG measures were unaffected; study reported ERPs in response to standard stimuli
Pekkonen et al. (160)	Haloperidol challenge 2 mg in 12 healthy volunteers	No effect on MMN	Study reported ERPs in response to standard stimuli
Ahveninen et al. (161)	5HT challenge using acute tryptophan depletion versus placebo control in 13 healthy volunteers	Delayed MMN latency	Study reported ERPs in response to standard stimuli
Umbricht et al. (131)	Single-blind, placebo-controlled psilocybin challenge (0.28 mg/kg) in 18 healthy volunteers and ketamine (0.9 mg/kg/h) in 20 healthy volunteers whilst performing a continuous performance task	Smaller MMN to frequency and duration deviants was correlated with stronger effects on the brief psychiatric rating scale during ketamine but not psilocybin	Study does not report MMN in the respective placebo conditions and did not report ERPs in response to standard stimuli
Leung et al. (162)	Double-blind, placebo-controlled crossover administration of bromocriptine (2.5 mg) or pergolide (0.1 mg) in 15 healthy volunteers	No effect on MMN, P1, N1, N2, and P3	Study reported ERPs in response to standard stimuli
Korostenskaja et al. (163)	Double-blind, placebo-controlled crossover administration of methylphenidate challenge (40 mg) in 13 healthy volunteers	No effect on MMN or N1; P2 amplitude reduction with methylphenidate	Study reported ERPs in response to standard stimuli
Leung et al. (164)	Double-blind, placebo-controlled crossover design following tyrosine/phenylalanine and/or tyrosine depletion intervention in 16 healthy volunteers	No effects on ERPs	Study reported ERPs in response to standard stimuli

In summary, the literature to date on the pharmacology of MMN generally reflects its obvious relationship with experience-dependent synaptic plasticity. The observed effects of ketamine, nicotine and cannabis provide support for the NMDAGluR-susceptibility of the system underlying MMN and therefore utility to schizophrenia research on MMN as they offer useful insights into the neurobiological processes that can influence or modulate MMN amplitude. However the memantine studies clearly caution that the relationship between perturbation of NMDAGluRs and MMN amplitude is not a simple one. There is insufficient information within most published studies to determine exactly how the various agents are altering the underlying processes with most reporting on difference waveforms only. Clarity regarding which elements of changed responsiveness are affected by drugs is particularly important to a thorough understanding of the process and these issues are discussed further in Section “Translation to Animal Models” below. Where possible it may be advantageous to add genotyping to pharmacology studies as it is well known that susceptibility to drug effects can be dependent on genetic profiles but of course the cost and sample size requirements are often prohibitive.

## Translation to Animal Models

Animal models have the potential to inform investigation of the physiological basis of MMN and potentially how it is disrupted in persons with schizophrenia. However, the primary issue of debate in animal models of MMN is to determine which components of the MMN process can be observed (and under which conditions they can be observed) in animals, there being some skepticism expressed in the past over whether the rodent brain exhibits “true MMN” (165). Debate over how exactly to measure MMN (see The Measurement of MMN – Is there an Optimal Paradigm with Which to Study MMN in Schizophrenia?) is therefore particularly relevant to animal work. Results from several different control designs are reviewed in this section and for ease of communication they are presented diagrammatically in Figure 1.

The presence of adaptation to repeated stimuli (or stimulus-specific adaptation, SSA) in A1 is well-described in the cat (8, 166), rat (167–170), and macaque (171). A1 neuron populations will adapt to repetitive stimuli and will exhibit relatively large responses to a rare deviant compared to a common standard. However, as reviewed in Section “The Measurement of MMN – Is there an Optimal Paradigm with Which to Study MMN in Schizophrenia?” evidence of “true MMN” must include something more than just adaptation effects. Terminology used is not consistent across animal and human studies so for simplicity we adopt the following nomenclature: oddball mismatch response (MMR) is used when referring to the difference between the response to a rare deviation and the regular (usually physically different) repeating sound; and controlled MMR is used to define evidence of contextual deviance detection when a study has included some kind of control for adaptation and physical characteristics of the stimuli (see Figure 1).

### What is the evidence for a “controlled MMR” in local field potentials?

Table 4 summarizes the animal studies that utilize oddball paradigms to test for evidence that a contextually deviant sound has been detected. The location of the recording electrode plays a large role in the findings of these studies, as epidural-placed electrodes receive input from a larger and more distributed network of cortical neurons than local field potential electrodes (LFP; measuring changes in synaptic potentials) or electrodes that register multi- or single-unit spike activity [MUA, SUA; see Ref. (165)]. The majority of studies using LFP recordings have searched for evidence of contextual deviance detection by recording from A1, but some also have recorded from the hippocampus (172–174). The majority of investigations using LFP or MUA (intra-cortical recordings), that also use many-standards or deviant-alone control, have not found any evidence for true MMR in the A1 (8, 167, 169, 171), whereas the majority of studies using *epidural* recordings and appropriate controls *have* found evidence for contextual deviance detection. This indicates that while adaptation to stimulus frequency/pitch does occur in the A1, the deviance-detection component of the MMN has not been identified in this region, and if it is present in the A1, it may be generated by more distributed networks than can only be observed using LFP recordings.

**Table 4:** **Summary of papers investigating mismatch responses in animal models**.

Reference	Species/strain	Recording type/location	Anesthesia	Control type	Oddball MMR finding	Controlled MMR finding	Effects of NMDAGluR antagonism
**SUBDURAL RECORDINGS**							
Ulanovsky et al. (8)	Cat	MUA in A1 and MGB	Xylazine/ketamine	None	Yes	N/A	
Ulanovsky et al. (166)	Cat	LFP in A1 and MGB	Xylazine/ketamine	None	Yes	N/A	
Fishman and Steinschneider (171)	Cat	LFP in A1	Awake	Many-standards	Yes	No	
Javitt et al. (130)	Macaque	LFP in A1	Awake	None	Yes	N/A	Infusions of PCP (5 and 10 μg) into the auditory cortex reduced oddball MMR
Ehrlichman et al. (172)	Mouse (DBA/2Hsd)	LFP in CA3	Awake	None	Yes	N/A	Ketamine (10 mg/kg) reduced oddball MMR
Ehrlichman et al. (173)	Mouse (C57/129Sv background)	LFP in CA3	Awake	None	Yes	N/A	
Farley et al. (169)	Rat (unknown strain)	LFP and MUA in A1	Awake	Many-standards	Yes	No	
Eriksson and Villa (167)	Rat (Long–Evans)	LFP in A1	Awake	Deviant-alone	Yes	No	
Imada et al. (175)	Rat (Long–Evans)	LFP in frontal and parietal cortices	Awake	Deviant-alone, standard-alone	Yes	Yes	
Taaseh et al. (170)	Rat (Sabra)	LFP and MUA in A1	Halothane	Many-standards, deviant-alone	Yes	Yes	
von der Behrens et al. (168)	Rat (Sprague–Dawley)	LFP and MUA in A1	Awake	None	Yes	N/A	
**EPIDURAL RECORDINGS**							
Pincze et al. (176)	Cat	Epidural above A1	Awake	None	Yes	N/A	
Umbricht et al. (177)	Mouse (C57/129)	Epidural above A1	Awake	Deviant-alone	Yes	No	
Jung et al. (178)	Rat (black hooded)	Epidural above A1	Awake	Many-standards	Yes	Yes	
Tikhonravov et al. (179)	Rat (Hannover–Wistar)	Epidural near A1	Pentobarbital sodium	Deviant-alone	Yes	Yes	High-dose (0.3 mg/kg), but not low-dose (0.1 mg/kg) MK-801 reduced oddball MMR
Tikhonravov et al. (180)	Rat (Hannover–Wistar)	Epidural near A1	Pentobarbital sodium	Deviant-alone	Yes	Yes	Low-dose (3 mg/kg) memantine increased oddball MMR, high-dose (10 mg/kg) reduced oddball MMR
Roger et al. (181)	Rat (Long–Evans)	Epidural above motor, parietal and anterior cingulate cortices	Awake	None	Yes	N/A	
Ahmed et al. (182)	Rat (Sprague–Dawley)	Epidural above A1	Urethane	Many-Standards	Yes	Yes	
Astikainen et al. (183)	Rat (Sprague–Dawley)	Epidural above A1	Urethane	Many-Standards	Yes	Yes	
Lazar and Metherate (184)	Rat (Sprague–Dawley)	Epidural above A1	Urethane/xylazine	Deviant-alone	Yes	No	
Ruusuvirta et al. (174)	Rat (Sprague–Dawley)	Epidural above A1/LFP in subiculum, DG, CA1	Urethane	None	Yes	N/A	
Astikainen et al. (185)	Rat (Wistar)	Epidural above A1	Urethane	None	Yes	N/A	
Nakamura et al. (186)	Rat (Wistar)	Epidural near A1	Awake	Many-Standards	Yes	Yes	
Ruusuvirta et al. (187)	Rat (Wistar)	Epidural above A1	Urethane	Deviant-alone	Yes	Yes	
Ruusuvirta et al. (188)	Rat (Wistar)	Epidural above A1	Urethane	None	Yes	N/A	

*A1, primary auditory cortex; CA1, CA1 region of hippocampus; DG, dentate gyrus; LFP, local field potentials; MGB, medial geniculate body of thalamus; MUA, multi-unit activity; SUA, single-unit activity*.

One of the first animal model studies (130) found larger responses to deviant sounds in an oddball paradigm originate in the supragranular layers (II–III) and are dependent upon normal functioning of the NMDAGluR (covered in Section “How do Pharmacological Manipulations Alter the MMN Process?”). However, Javitt et al. did not utilize a many-standard control and in a more recent study that did (171), larger responses *were not* observed to the deviant compared to the same tone in the many-standards sequence (no evidence of true MMR). The many-standards control in this study may have overestimated adaptation, however, as the deviant was presented at a probability of 10% in the oddball sequence, and the tones used for the many-standards control were presented at a maximum rate of 5% (171).

There are, to our knowledge, only two studies thus far utilizing LFP recording that have found some evidence for what might be considered controlled MMR in the rat brain (170, 175). Imada et al. (175), using a frequency deviant paradigm, found that the difference between the ERPs to deviant and standard stimuli was significantly elevated from 0 in an oddball MMR. However as a control they included separate sequences preserving the timing of standard sounds (without deviants) and deviant sounds (without standards) from the original oddball sequence. The ERP to deviant-alone and the standard-alone did not differ (175). According to the authors, this indicates that there was a statistically significant change in the response to the deviant stimuli in the oddball measure, over and above adaptation, which may hint at the presence of true MMR, albeit one that is not large enough to produce a statistically significant difference in responses to the oddball deviant compared to the deviant-alone. Taaseh et al. (170) utilized both LFP and MUA recording and found that the response to the oddball deviant tones did not exceed the response to the same tone in a many-standards control sequence (their *diverse-broad* sequence). However, the response to the deviant sound *was* larger than the many-standards control in some recordings (although not at a population level), indicating that perhaps a subset of neurons do exhibit sensitivity to the contextual deviance of the sound (170). In addition, their thorough characterization of adaptation effects was used to generate a model which predicted that if only adaptation were occurring, the response to the deviant would be smaller than the response to the same tone in the many-standards control. However, these responses did not differ significantly, suggesting (albeit rather indirectly) that contextual deviance detection is occurring in the auditory cortex. There is therefore some evidence that recognition of contextual deviance occurs in the rat A1. Such hints of response to contextual aberrance may represent the first step in a cascade that results in full MMN responses (170). This study, however, did not identify the significantly elevated response to the deviant compared to the control that is similar to the MMN typically seen in humans– a pattern only observed in animal models when recorded from larger brain volumes (165).

### What is the evidence for a “controlled MMR” in epidural recordings?

Studies using epidural recordings in animals (mainly rats) use electrodes implanted in the skull or sitting on the dura over the auditory cortex (or close to the auditory cortex) to record ERPs elicited during oddball sequences. Table 2 summarizes these studies. Of these, four use the many-standards control. Ahmed et al. (182) used speech sounds as stimuli, and found larger responses to the deviant sound/ba/in an oddball sequence compared to the same sound presented at the same rate in a many-standards control in anesthetized rats (182). Nakamura et al. (186) and Jung et al. (178) both investigated responses to frequency/pitch deviants in awake rats, and found larger responses to deviant stimuli compared to those in the many-standards control sequence (178, 186). Astikainen et al. (183) examined responses to frequency deviants in *anesthetized* rats and found evidence of deviance detection when the deviant was a high frequency (4.2 kHz) stimulus, not a low frequency stimulus (3.8 kHz), as was also found in Nakamura et al. (3.6 Vs. 2.5 kHz) (183). Nakamura et al. also investigated the effect of duration deviants and identified larger responses to long (but not short) duration deviants compared to the same tones in the many-standards control sequence (186). Therefore all four studies using the many-standards control have indeed observed a response reminiscent of controlled MMR.

Although all these studies all report deviance detection in the rat brain, the morphology of the ERPs differs between each of the studies, with the most dramatic difference in ERPs being observed between the awake recordings (178, 186) and the anesthetized recordings (182, 183). Awake ERPs comprise 3–4 negative and positive components, and the anesthetized recordings feature only one large positive component. The polarity of the deviance-detection effects is also different, with negative deflections in response to the deviant in the awake animals, and positive deflections in anesthetized animals. Such trends can be similarly observed in other studies (Table 4), with the majority differences between the deviant and the standard being positive in anesthetized rats and negative in awake rats. These findings illustrate that deviance-detection need not be a response that exactly mimics the human MMN (insofar as being of the same polarity). Indeed human mismatch responses (MMR) change from negative to positive in polarity when the recording location is moved from fronto-centrally sites to the mastoid when recorded using a nose reference (189).

The remainder of studies examining epidural ERPs in oddball paradigms use either the deviant-alone to control for adaptation, or use no adaptation control at all. Although the deviant-alone sequence may overestimate the contribution of adaptation (165), three of the five studies utilizing this control sequence find evidence for controlled MMR recorded from epidural electrodes (179, 180, 187). Of those epidural studies that did not implement a control for adaptation, several did not use simple frequency deviants (which are highly predisposed to be affected by adaptation mechanisms in the A1), but rather used more complex deviants that presumably elicited responses that would not be so readily perturbed by neural adaptation. For instance, Roger et al. (181) measured responses to duration deviants [which are not affected by adaptation in the A1 to the same degree as frequency deviants; (169)], and found larger responses to duration deviants (181). In addition, Ruusuvirta et al. (174) examined responses to duration deviants in combination with stimulus onset asynchrony deviants, and found evidence for larger deviant responses (compared to standards) only in the condition in which the deviant occurred earlier than expected and deviated in duration, indicating a possible threshold level for deviance that needs to be reached before a deviance-detection-like response iselicited (174). However, without the use of a many-standards (or even a deviant-alone) control, it remains difficult to conclude that such responses are completely independent of adaptation effects. Although tone duration is coded in a different way to the frequency, and previous studies have demonstrated that adaptation mechanisms for duration are not as robust as they are for frequency (169), this was examined in the auditory cortex only, and it cannot be ruled out that another region (or network of regions) adapts to stimulus duration in the same way as the auditory cortex does for frequency. These studies show that when using methods that detect shifts in potential at a network-level (i.e., a large spatial scale), signs of human-like MMR (independent of adaptation) are evident in animals.

### How does animal research augment human research on MMN?

Animal models are an ideal tool with which to investigate the underlying neurobiology of MMR because of the ability to perform more invasive and selective neurobiological manipulations (e.g., drug microinjection to specific regions). In addition, the comprehensive genetic toolkit available for mouse models will enable researchers to determine the role of specific genes and neural populations in the generation of MMR using transgenic animals and optogenetics. With a consensus emerging regarding the ideal recording method (epidural) and the current preferred control to use (many-standards) to examine MMR (adaptation and deviance detection) in animal models, the time is ripe for further studies examining the neurobiology of these elements of MMR, using pharmacological, developmental, and genetic manipulations. Unfortunately, few studies implement a control for adaptation effects (or if they do, do not adequately report drug effects on these), and it is therefore difficult to determine whether or not the agents given affect adaptation, deviance-detection, or both. The preferable way to investigate the pharmacology of MMN in animal models would be test different manipulations in a model that exhibits both adaptation and deviance detection [e.g., Ref. (178)], and to compare how different agents affect the responses to standards, deviants, and control stimuli; with the difference between the control and standard representing adaptation and the difference between the deviant and the control representing MMR and the effects of drug interventions on these two separate components can be examined. To our knowledge, no studies have tested such a model thus far.

Studies in rats and mice (like those on the macaque described above) have been used to study the role of the NMDAGluR system in MMN. These studies, in contrast to Javitt et al. (130), suggest that perturbations in NMDAR signaling can also alter responses to standard stimuli. In the absence of a control for adaptation effects, Ehrlichman et al. found that ketamine (a non-competitive NMDAGluR antagonist) concurrently *increased* the response to the standard (which was small to start with) and *reduced* the response to the deviant, albeit not to a significant degree, in awake mice (172). Tikhonravov et al. used a low (0.1 mg/kg) and high (0.3 mg/kg) dose of intraperitoneal MK-801 to examine the effects of NMDAGluR perturbation on responses to oddball stimuli, in addition to a deviant-alone control in pentobarbital-anesthetized rats (179). Responses to the deviant were more positive than the responses to the standard for the saline condition and were relatively unchanged by the low-dose of MK-801. However, the high-dose of MK-801 lowered the response to the deviant and increased the response to the standard, effectively reversing the polarity of the oddball effect. The degree to which MK-801 altered the *magnitude* of the difference between the oddball deviant and the deviant-alone was not reported. However, the period over which the deviant was significantly different from the deviant-alone was reported: there was a positive deflection in response to the deviant (relative to deviant-alone) in saline-treated animals (indicating deviance detection), which was absent after low-dose MK-801 and reversed in polarity after high-dose MK-801 (179). The finding that low-dose MK-801 can reduce the difference between the oddball deviant and deviant-alone, without a change in the differences between deviant and standard could indicate that this dose can preferentially disrupt deviance detection, while sparing adaptation. However, the high-dose of MK-801 not only disrupted both deviance detection and adaptation but reversed the polarity of these changes. Such reversals, caused by an increase in the response to the standard and a decrease in the response to the deviant, are similar to those found after ketamine by Ehlrichman et al.

In a second study, Tikhonravov et al. investigated the effects of a low (3 mg/kg) and a high (10 mg/kg) dose of memantine on responses to oddball stimuli and deviant-alone stimuli in anesthetized rats (180). Like MK-801, memantine is an uncompetitive NMDAGluR antagonist, but unlike MK-801, is a very low-affinity antagonist and has potential as a cognitive enhancer [(190) see also discussion of memantine in Section “How do Pharmacological Manipulations Alter the MMN Process?”]. Similar to previous studies in anesthetized rats, the oddball MMR was positive in saline-treated rats. The low-dose of memantine resulted in a significant increase in the response to the deviant and the oddball MMR was significantly prolonged compared to the saline group. The high-dose of memantine, on the other hand, reduced the time over which the oddball MMR was significantly different from 0 and reversed the late phase of the difference waveform, possibly due to an increase in the response to the standard (180). With regard to deviance-detection-specific changes, the deviant was more positive than the deviant-alone in saline-treated rats, an effect that was prolonged in the low-dose memantine group. In rats treated with the high-dose of memantine, however, no deviance-detection response was observed. These findings indicate that low-dose memantine potentiates the deviance-detection response, with no significant effect on the adaptation response, but that high-dose memantine acts similarly to high-dose MK-801, affecting both adaptation and deviance detection and reversing the polarity of the oddball MMR.

Overall, these pharmacological studies suggest that NMDAGluR-antagonists act in a dose-dependent fashion, with low-dose/low-affinity antagonists facilitating deviance detection (by increasing the response to the deviant), then as NMDAGluR perturbations are increased with low-dose/high affinity antagonists, deviance detection is inhibited while adaptation is spared. However, with high-doses of high affinity blockers (ketamine and MK-801), the response to the standard is increased, thus indicating impaired adaptation. The selective impairment of deviance detection and not adaptation after MK-801 is also highlighted in (169), where it was reported that subcutaneous MK-801 (maximum dose, 0.1 mg/kg) reduced responses to both the deviant and the standard together, while preserving the difference between them, indicating that this dose *did not* affect neural adaptation in A1, similar to findings in the study in which the same drug was given intraperitoneally (179). These studies indicate that the mechanism by which NMDAGluR-antagonists may reduce the MMN may be rather complex, with the dose and affinity of the antagonist interacting with adaptation and deviance-detection resulting in varied effects on these elements of the MMN. This animal work highlights a complexity in the role of NMDAGluRs in the generation of MMRs that was not discovered in human studies using NMDAGluR-antagonists, most likely due to the smaller dose-range, smaller sample size, and lack of consistent reporting of the response to standards seen in the human work. This animal work therefore highlights the need to examine a larger dose-range, as well as the need for consistent reporting of both standard and deviant responses in human pharmacological MMN studies.

Several of the previously mentioned animal pharmacological studies have similar weaknesses highlighted for the human studies. While (179, 180) used a control for adaptation (the deviant-alone control), they did not report directly on the magnitude of responses to the standard, the deviant and the deviant-alone control, thus making the interpretation of the mechanisms affected by pharmacological agents very problematic. While these animal model investigations are still in their infancy, the promise of such models is far-reaching. Future investigations will be able to focus on a range of doses of NMDAGluR-antagonists in paradigms in which adaptation and deviance detection are well-described, to further explore the role of NMDAGluR signaling in both of these components. In addition, NMDAGluR-antagonists can be infused directly into regions of interests [as performed by Javitt et al. (130)], to determine where NMDAGluR signaling is important for MMR. Further, high-doses of muscimol [a Gamma-Amino Butyric Acid (A) agonist] can be infused to completely inactivate regions to determine their contribution to deviance detection. For example (174) found evidence of MMR in rat hippocampus – the degree to which these contribute to epidural-recorded MMR can be determined by infusing muscimol into selected hippocampal regions. In addition, animal models of schizophrenia-like reductions in MMN could possibly be developed. (173) used transgenic mice heterozygous for *neuregulin 1* (*nrg1*; an hypothesized schizophrenia-susceptibility gene) and found a reversal in polarity of the oddball MMR in *nrg1* mutants compared to wild type mice. However, this study did not adopt a control for adaptation effects, so it is unknown in *nrg1* plays a role in adaptation or adaptation-independent deviance-detection. Experiments in our lab are currently underway to determine the effect of maternal immune activation on MMR (both adaptation and deviance detection) in rats. Maternal immune activation is a risk factor for schizophrenia and when modeled in rats and mice, is associated with numerous schizophrenia-like behavioral and neurodevelopmental outcomes, particularly those related to NMDAGluR-dysfunction (191–193). This model therefore may also exhibit schizophrenia-like changes in MMR and could be used as a potential experimental platform to examine the pathology underlying schizophrenia-like reductions in the mismatch response, and potential treatments for such alterations.

## Conclusion

The above review supports several conclusions regarding the potential of MMN as a tool to study the biological processes taking place in those with (and potentially those at-risk for) schizophrenia.

### Measurement and reporting of MMN

There are pros and cons to any experimental design and it is the authors’ opinion that there is no optimal design for use in schizophrenia studies. To recommend an optimal design, while improving consistency in the literature, would come at the expense of the unique contributions that can be made by novel designs [see Ref. (24) for review] that can enrich our understanding of the perceptual inference within the MMN process and how gain in this signal is controlled. Furthermore, study design is often limited by mundane and yet important considerations of test duration that could render particular paradigms less feasible. However, two recommendations arise from this review. The first is that authors and journals facilitate a closer adherence to publication standards. A failing of the current literature is that a large number of studies (including some by our group) do not adhere to recommended publication standards for ERP research which stipulates the display of the original ERPs from which difference waveforms have been derived (194). By comparing research findings across species and drug studies (as above) the importance of at least reporting standard and deviant ERPs becomes clear. Secondly, while the traditional oddball paradigm provides a robust measure of the reduction in MMN amplitude in schizophrenia, protocols designed to identify constituents of the MMN process (or oddball combined with rigorous controls) introduce the capacity to begin disentangling which components of the process are in fact compromised. This advantage becomes crucial when attempting the translation of this research into animal models (see Translation to Animal Models) and arguably also to pharmacological studies (see How do Pharmacological Manipulations Alter the MMN Process?). Where feasible, future studies should consider the advantage of designs (or analysis techniques) that facilitate the differentiation between adaption effects and something more akin to true contextual deviance detection.

### Vulnerability to schizophrenia

Our review of the literature to date indicates limited support for small MMN conferring some vulnerability to schizophrenia but we consider this an open question best addressed by large longitudinal studies. It is the authors’ opinion that there is much to gained by continued efforts to understand factors that influence MMN size (using novel designs, pharmacological, and animal research) in parallel to such efforts.

### Pharmacology and animal models of MMN

It seems clear from the pharmacological studies (in animals and humans) that adaptation and sensitivity to contextual deviance may show differential effects in the presence of compromised NMDAGluR function. Pharmacological, like animal studies, require indices of both to be informative about the effect of agents on the MMN process. Similar to schizophrenia studies, animal, and pharmacological studies inconsistently report on standard and deviant effects. While our review of pharmacological studies is more supportive of some agents (e.g., glutamatergic and cholinergic) than others (e.g., monoamines), our knowledge of influences on perceptual inference and learning underlying MMN continues to grow and challenge existing models [e.g., Ref. (98, 195)] and it remains possible that future paradigms may be sensitive to agents that current paradigms are not. In other areas of learning, animal models and pharmacology have provided great insight into schizophrenia [associative learning (196)] and there is good reason to suppose that this will also be true of MMN research as well as offering the potential to examine commonalities in underlying pathology. While there are many challenges in translating the potential of MMN in elucidating the pathophysiology of the schizophrenic illness, we believe the current state of research encourages scientists to pursue the many numerous potentially fruitful avenues available to achieve this goal.

## Author Contributions

Juanita Todd, – lead writer and overall editor. Primary contributions Sections “The Potential – Why is there so Much Interest in MMN in Schizophrenia?” “The Measurement of MMN – Is there an Optimal Paradigm with Which to Study MMN in Schizophrenia?” “How do Pharmacological Manipulations Alter the MMN Process?” and “Conclusion.” Lauren Harms – Primary contribution Section “Translation to Animal Models.” Reference manager. Patricia T. Michie – Primary contributions Sections “The Measurement of MMN – Is there an Optimal Paradigm with Which to Study MMN in Schizophrenia?” and “Does MMN-Reduction Indicate Vulnerability to Schizophrenia?” Ulrich Schall – co-writer of all sections and preparation of Tables 1–3.

## Conflict of Interest Statement

The authors declare that the research was conducted in the absence of any commercial or financial relationships that could be construed as a potential conflict of interest.

## Footnotes

^1^A somewhat puzzling result in the Jessen et al. study, is that patient MMN amplitude was not significantly reduced in contrast to relatives.
